# Community-associated Methicillin-resistant *Staphylococcus aureus* in Pediatric Patients

**DOI:** 10.3201/eid1106.050142

**Published:** 2005-06

**Authors:** Theresa J. Ochoa, John Mohr, Audrey Wanger, James R. Murphy, Gloria P. Heresi

**Affiliations:** * University of Texas Health Science Center at Houston, Houston, Texas, USA;; † Memorial Hermann Hospital, Houston, Texas, USA

**Keywords:** Methicillin Resistance, Staphylococcus, Pediatric Infectious Diseases/vaccines, AOM, bacterial respiratory infection, S. pneumoniae, antibiotic treatment, antibiotic tr, infection, Antibiotic resistance

## Abstract

Community-associated methicillin-resistant *Staphylococcus aureus* (CA-MRSA) infections increased from 2000 to 2003 in hospitalized pediatric patients in Houston. CA-MRSA was associated with greater illness than was infection with methicillin-susceptible strains. Children with CA-MRSA were younger and mostly African American. Of MRSA isolates, 4.5% had the inducible macrolide-lincosamide-streptogramin B phenotype.

Community-associated methicillin-resistant *Staphylococcus aureus* (CA-MRSA) infection in children is an increasing problem (1,2). However, we do not know whether CA-MRSA and the historically more common community-associated methicillin-susceptible *Staphylococcus aureus* (CA-MSSA) have similar pathogenesis and cause similar illness (3–5). In Houston, CA-MSSA infections were reported initially to be more severe than CA-MRSA infections (3), but further reports stated the opposite (4,5). Our clinical impression was that CA-MRSA infections were becoming more frequent and were more severe than CA-MSSA infections. To test the validity of our clinical impression, we performed a retrospective chart review of hospitalized pediatric patients with *S. aureus* infections during a 3-year interval. We determined prevalence, clinical characteristics, susceptibility patterns, and empiric antimicrobial regimens for CA-MRSA and CA-MSSA.

## The Study

We performed a retrospective chart review of pediatric patients (≤18 years of age) who were admitted to Memorial Hermann Children's Hospital, Houston, Texas, in a 36-month period (July 2000 to June 2001 and January 2002 to December 2003; we excluded the second semester of 2001 from the analysis because the hospital and the microbiology laboratory were temporarily closed in July 2001). A laboratory report of isolation of *S. aureus* from an inpatient qualified the person as a candidate. From these candidates, patients with underlying illness predisposing to frequent hospitalization (immunodeficiency, cystic fibrosis, chronic renal failure, malignancy) and patients who had been previously hospitalized or underwent surgery within 3 months before *S. aureus* isolation were excluded. Patients from the neonatal intensive care unit and patients with mixed cultures were also excluded. For the remaining patients (N = 239), community-associated *S. aureus* was defined as the isolation of *S. aureus* from a culture obtained within 72 h of admission. Antimicrobial resistance testing was performed by broth microdilution MIC method by the Clinical Microbiology Laboratory (Pasco, Becton Dickinson, Sparks, MD, USA).

Clinical and Laboratory Standards Institute (CLSI) (formerly NCCLS) standards and guidelines were used to interpret MICs for clindamycin, erythromycin, gentamicin, linezolid, minocycline, oxacillin, fluoroquinolones (ciprofloxacin, levofloxacin, gatifloxacin), rifampin, trimethoprim/sulfamethoxazole (TMP/SMX), and vancomycin. For MRSA isolates that were erythromycin resistant and clindamycin susceptible, inducible macrolide-lincosamide-streptogramin B (MLSB) resistance was determined by the disk diffusion method (6). Demographic and clinical characteristics between CA-MRSA and CA-MSSA were compared by Student *t* test or Wilcoxon signed rank for continuous variables and chi-square/Yates correction or Fischer exact test for categorical variables.

From 2000 to 2003, CA-MRSA accounted for 67% (159/239) of community-associated *S. aureus* infections in hospitalized pediatric patients (56% in 2000–2001, 57% in 2002, and 78% in 2003, p<0.01 for trend). Patients with CA-MRSA infections were significantly younger and more likely to be African American than patients with CA-MSSA infections, which is consistent with results from a previous study (3). Patients with CA-MRSA tended to have longer duration of bacteremia and significantly more surgical interventions (incision, aspiration, drainage, or débridement) (Table 1). Both groups had similar duration of hospitalization, intensive care unit treatment, proportion of positive blood culture, peripheral leukocyte counts, and erythrocyte sedimentation rates at admission (data not shown).

**Table 1:** Demographic and clinical characteristics of hospitalized pediatric patients with CA-MRSA and CA-MSSA infections*

Demographic data	No. MRSA (n = 159), (%)	No. MSSA (n = 80), (%)	p value
Age, median (range)	1.6 y (1.5 mo–17.9 y)	2.6 y (2 mo–17.7 y)	<0.05
Female sex	86 (54.0)	38 (47.5)	NS
Ethnicity			
African American	75 (47.1)	27 (33.7)	<0.05
Hispanic	52 (32.7)	32 (40.0)	NS
White	21 (13.2)	14 (17.5)	NS
Other	11 (6.9)	7 (8.7)	NS
Clinical data			NS
Duration bacteremia (d)†, mean ± SD	2.4 ± 1.8	1.1 ± 0.3	0.06
Surgical intervention	140 (88.1)	57 (70.4)	<0.01
Hospital days, median (range)	3 (1–53)	4 (1–38)	NS
Intensive care‡	13 (8.2)	10 (12.5)	NS

*CA, community-associated; MRSA, methicillin-resistant *Staphylococcus aureus*; MSSA, methicillin-susceptible *S. aureus*; NS, not significant; SD, standard deviation. †Positive blood cultures: 7 MRSA and 9 MSSA. ‡No. patients who required treatment in the pediatric intensive care unit.

CA-MRSA infections were seen more frequently with abscesses and complicated pneumonias (Table 2). The locations of the abscesses were similar in both groups; the most common sites were the extremities, gluteal, and perirectal areas. Among deep abscesses, 2 mediastinal and 1 retropharyngeal abscess were CA-MRSA, and 1 retropharyngeal abscess was CA-MSSA. Among patients with pneumonia, 12 of 17 CA-MRSA were complicated (9 empyemas and 3 pneumatocele/pneumothorax) versus 2 of 13 CA-MSSA (1 empyema and 1 pneumatocele). Only 2 patients had a documented viral pneumonia before the *S. aureus* pneumonia. Among patients with osteoarticular infections, both groups had similar involvement and complications. In the CA-MRSA group (10 patients) were 7 osteomyelitis, 5 septic arthritis, 1 myositis, 1 deep venous thrombosis, and 4 bacteremia cases. In the CA-MSSA group (8 patients) were 8 osteomyelitis, 2 septic arthritis, 3 myositis, 1 deep venous thrombosis, and 4 bacteremia cases.

**Table 2:** Site of infection with CA-MRSA or CA-MSSA*

Site	No. MRSA (n = 159), (%)	No. MSSA (n = 80), (%)	p value
Abscess	80 (50.3)	23 (28.7)	<0.01
Lymphadenitis	35 (22.0)	13 (16.2)	NS
Pneumonia	17 (10.7)	13 (16.2)	NS
Complicated pneumonia†	12/17 (70.6)	2/13 (15.4)	<0.01
Cellulitis	12 (7.5)	8 (10.0)	NS
Osteoarticular‡	10 (6.3)	8 (10.0)	NS
Other§	5 (3.1)	15 (18.7)	ND

*CA, community-associated; MRSA, methicillin-resistant *Staphylococcus aureus*; MSSA, methicillin-susceptible *S. aureus*; NS, not significant; ND, not done (various diagnosis grouped). †Empyema, necrotizing pneumonia, pneumatocele, or pneumothorax. ‡Osteomyelitis or septic arthritis. §Sinusitis, preseptal and septal cellulitis, retropharyngeal and mediastinal abscess, urinary tract infection, toxic shock syndrome, isolated bacteremia.

CA-MRSA isolates were more likely to be resistant to erythromycin (92% vs. 45%, p<0.01) and fluoroquinolones (16% vs. 4%, p<0.01). Resistance to clindamycin was 5% in both groups. All CA-MRSA and CA-MSSA isolates were susceptible to gentamicin, linezolid, minocycline, rifampin, TMP/SMX, and vancomycin. A total of 3 (4.5%) of 66 CA-MRSA isolates had the inducible MLSB-resistant phenotype. Clindamycin was the most commonly used antimicrobial drug. It was the initial empiric treatment for 60% of both CA-MRSA and CA-MSSA infections when used alone or in combination with other antimicrobial drugs. The use of clindamycin increased over time (32% in 2001, 54% in 2002, and 66% in 2003, p<0.001). The empiric use of vancomycin was more frequent in the CA-MRSA group (25% vs. 12%, p<0.05) but did not increase over time. Nafcillin use was similar in both groups (8% vs. 11%). For 16% of the CA-MRSA cases, empiric therapy was with an agent to which the infecting isolate was later found not to be susceptible in vitro, regardless of the clinical outcome.

## Conclusions

CA-MRSA is seen with increasing frequency in Houston; it is a more severe infection with more frequent serious complications, compared to CA-MSSA. The increasing frequency of severe *S. aureus* infection requires reassessing regimens of empiric therapy delivered on admission and added emphasis to timely and appropriate acquisition of specimens for culture.

Since 2000, rates of pediatric CA-MRSA in our hospital have increased from 56% to 78%; these infections are associated with greater illness, especially empyema and necrotizing pneumonia, compared to CA-MSSA infections. A similar increase in MRSA frequency and severity has been reported from another pediatric hospital in Houston (4,5). Of all CA-MRSA isolates reported from that hospital, 94% are of the same clone (7). The similar characteristics and rates across institutions support the hypothesis that CA-MRSA in Houston are related, but molecular genetic analysis of our strains would be necessary to confirm this hypothesis.

Emerging CA-MRSA is a global problem (1,2). For some regions, direct evidence shows an association between clonality of CA-MRSA and severity (5,8–10). This association seems to be related to specific virulence factors, such as the Panton-Valentine leukocidin, among others (10,11). In a recent study from Houston, strains carrying the *pvl* gene were associated with severe staphylococcal sepsis in adolescents (5) and with CA-MRSA musculoskeletal infection in children (8). The presence of the *pvl* gene may be related to an increased likelihood of complications in children with *S. aureus* infections. The present study lacks molecular genetic analysis of the strains to support this hypothesis.

Treatment of MRSA infections is challenging. Empiric treatment usually includes the use of clindamycin or vancomycin (2,4). MRSA strains that are clindamycin-susceptible but erythromycin-resistant may have the in vitro inducible MLSB-resistance phenotype with potential for treatment failure (12–14). Rates of inducible MLSB resistance among pediatric MRSA isolates vary widely. Our results are similar to those from previous studies from Houston (2%–8% inducible MLSB resistance) (3,4) and different from reports from cities such as Baltimore (43%) (13) and Chicago (94%) (12). Awareness of local resistance patterns is required to select adequate empiric therapy. Trends in clindamycin use could indicate physician awareness of MRSA resistance patterns. The increasing penetration of CA-MRSA in the community requires disseminating information to primary care providers about the potential severity of this infection, methods for rapid and accurate diagnosis, and need to rapidly implement appropriate empiric and definitive treatment regimens.
